# Direct causality measures unravel complex networks of cardiovascular dynamics and their modifications with postural stress

**DOI:** 10.1371/journal.pcbi.1014075

**Published:** 2026-03-18

**Authors:** Chiara Barà, Laura Sparacino, Luca Faes, Michal Javorka

**Affiliations:** 1 Department of Engineering, University of Palermo, Palermo, Italy; 2 Institute of Intelligent Industrial Technologies and Systems for Advanced Manufacturing, National Research Council, Milan, Italy; 3 Department of Physiology, Comenius University in Bratislava, Jessenius Faculty of Medicine in Martin, Martin, Slovak Republic; Radboud Universiteit, NETHERLANDS, KINGDOM OF THE

## Abstract

This study provides a novel network perspective on the spontaneous short-term regulatory mechanisms underlying cardiovascular and cardiorespiratory interactions during different physiological states. The direct causality measure of conditional transfer entropy was estimated employing linear model-based and nonlinear model-free approaches and applied to the network of beat-to-beat heart period, arterial pressure, respiration, and arterial compliance variability series assessed in thirty-nine young healthy subjects monitored in the supine resting state and during orthostatic stress. The network analysis retrieved well-known regulatory mechanisms significant in most of the subjects, such as the tilt-induced decreased respiratory sinus arrhythmia and increased baroreflex, and uncovered less explored interaction pathways involving compliance. Specifically, we found a decreased effect of heart period and arterial pressure on compliance, as well as a stronger causal influence from compliance to arterial pressure and a decreased influence of compliance on respiration. The joint use of model-based and model-free approaches allowed us to infer the linear or nonlinear nature of these interactions. Our study advocates the main role played by arterial compliance into the intricate hank of cardiovascular interactions, and documents the need to employ direct causality measures to infer the many complex mechanisms generating short-term cardiovascular oscillations.

## Author summary

Short-term regulatory mechanisms involving well-known cardiovascular parameters such as heart period, respiration and arterial pressure have been widely investigated in data-driven cardiovascular research. Nevertheless, the way they produce and buffer cardiovascular variability in different physiological states is not fully understood, especially in contexts where the effects of unobserved variables may play a key role. For instance, the causal interplay between arterial compliance variability and both heart rate and arterial pressure variabilities has never been explored from a network physiology perspective, and the mutual effects between these variables remain poorly understood. To fill this gap, we propose a thorough investigation of a physiological network comprising multiple cardiovascular variables, including the less explored arterial compliance beat-to-beat time series, in young healthy subjects monitored in the resting state and during postural stress. We provide novel insights into the characterization of link-specific physiological mechanisms through conditional causality measures estimated using both linear model-based and model-free approaches. We highlight the remarkable role of arterial compliance in governing cardiovascular variability during the supine rest and orthostatic challenge.

## Introduction

In the holistic view of Network Physiology, the human organism is seen as an integrated network constituted by multiple organ systems, each characterized by its own structural organization and functional complexity, which continuously interact to coordinate their functions, generate distinct physiological states in response to internal, external and pathological perturbations, and maintain homeostasis [1–3]. To investigate the coordination and interactions among diverse biological systems and subsystems, data-driven methods for network inference can be exploited to build network models from sets of observed multivariate time series describing the activity of the network nodes [4]. Such models are usually represented as graphs, where nodes represent different physiological subsystems or districts, and links map functional dependencies between these systems. Straightforward examples involve the well-known cardiovascular interactions between the heart rate and arterial pressure variabilities [5], as well as the tangled coordination between the cardiac and the respiratory subsystems [6]. Cardiovascular and cardiorespiratory interactions, which reflect the modulation of heart rate, arterial pressure and respiratory variabilities [7], have been largely studied, with the aim of disentangling both autonomic regulation mechanisms and mechanical effects occurring in diverse physiological states and conditions [8,9].

As the activity measured at the nodes of physiological networks results from a big variety of mechanisms, an appropriate characterization of these mechanisms would require the involvement of many variables: cardiovascular and cardiorespiratory loops represent only a portion of a more complex and wider system. While heart rate, arterial pressure and respiration still remain the most studied [7], advancements in the beat-to-beat analysis of physiological variables have recently emerged. For instance, little is known about the short-term variability nature of arterial compliance, a cardiovascular variable characterizing mechanical and structural properties of the arteries [10,11]. Arterial compliance variability is expected to be directly affected by the sympathetic nervous system, and mostly indirectly by vagal activity in different patho-physiological conditions, as well as by heart rate, blood pressure and respiratory variabilities [11–13]. Investigating how this parameter behaves within complex physiological networks is essential to characterize its role for physiological research and clinical purposes. Furthermore, physiological mechanisms are challenged by a number of stressors, e.g., postural stress induces a reorganization of cardiovascular oscillations and of their coupling related to the shift in the sympatho-vagal balance towards sympathetic activation and parasympathetic withdrawal [14,15]. Hence, probing the investigated network after its modification due to a given stressor is of remarkable importance to characterize the type and modalities of network adaptation.

From a methodological viewpoint, noninvasive analysis of spontaneous oscillations of physiological variables has typically been performed by multivariate time series analysis. Classical linear and nonlinear approaches defined in the time, frequency, and information-theoretic domains, such as those based on cross-correlation analysis [16], coherence analysis [16,17], and entropy measures [7,18], have been used to describe the system properties captured by network models. However, traditional methods are overall limited by their intrinsic pairwise formulation (i.e., only two network nodes are taken into account), thus neglecting potential confounding effects due to non-involved variables [19]. From the physiological point of view, these approaches are generally noncausal (i.e., measures are symmetric), meaning that they do not allow identification of the direction of interaction between the considered physiological signals. Nevertheless, understanding the direction of relationships between physiological subsystems from the analysis of spontaneous cardiovascular oscillations is of utmost importance, since it allows a noninvasive, detailed comprehension of physiological regulatory mechanisms [20]. In this context, past studies have been focused only on specific network links, such as the cardiovascular or cardiorespiratory closed-loops with feedforward and feedback mechanisms describing the pairwise interplay between the heart and vascular or respiratory systems, respectively [7,18,20]. Indeed, various time series analysis techniques have been developed for the quantification of these specific physiological coupling mechanisms, e.g., the baroreflex sensitivity as a measure of reflex autonomic control of the cardiovascular system [17] or the amplitude of respiratory sinus arrhythmia as an index of cardiac vagal tone [20]. Whilst recent efforts have been oriented to capture the complex dynamics involving three or more processes [18,19,21,22], studies that investigate and interpret the role of specific causal links among multiple physiological nodes in an exhaustive way are limited in the analysis of cardiovascular oscillations. Among these approaches, methodologies exploiting conditional causality approaches in multivariate contexts would allow to estimate the strength of the causal coupling between two given nodes canceling out the influence of the rest of the network.

In this work, we start from the hypothesis that arterial compliance may play a valuable role in the physiological mechanisms governing complex beat-to-beat cardiovascular regulation during different states. Therefore, exploiting sophisticated measures which allow to carry out a systematic analysis of direct causal interactions in physiological systems, we choose to examine a network comprising four variables of physiological and clinical interest. Our aim is to probe noninvasively the joint spontaneous beat-to-beat variability of the main cardiovascular and respiratory parameters, i.e., heart period, arterial pressure and respiration, including the novel arterial compliance time series, in the supine resting state and in response to the homeostatic alterations caused by the postural stress. In pursuit of this goal, we exploit diverse methodologies, namely parametric [23] and non-parametric [24] conditional causality approaches designed to disentangle putative linear and nonlinear dynamics, respectively. We reconstruct the structure of the investigated network in terms of presence/absence of direct links between pairs of variables. The physiological relevance and meaning of each of the observed interactions is first statistically validated and then accurately discussed to either confirm or confute previous results, but also to provide novel insights on the complex mechanisms governing the cardiovascular, cardiorespiratory, and vascular-respiratory interplays. To our current knowledge, our work represents the first attempt to exhaustively describe a complex network of physiological variables including the beat-to-beat arterial compliance time series, employing sophisticated multivariate measures of dynamical interaction.

## Materials and methods

### Ethics statement

The study was performed according to the Declaration of Helsinki and it was approved by the ethical committee of the Jessenius Faculty of Medicine, Comenius University of Bratislava [11,25,26]. All subjects or their legal representatives, in participants under 18 years of age, gave written informed consent before the examination.

### Study protocol

The cohort study comprised *39* young and healthy Caucasians (22 women, 17 men; median age: 18.7 yr) [11,25,26]. Subjects were positioned on a motorized head-up tilt table with a foot support, and were asked to avoid unnecessary speaking and moving during the measurement. The study protocol consisted of two consecutive phases: supine rest (REST, 15 min), and head-up tilt (HUT, the subject was tilted to 45 degrees for 8 min to evoke mild orthostatic stress). All subjects breathed spontaneously without any effort to control breathing rate or tidal volume.

### Data acquisition

**Fig 1:**
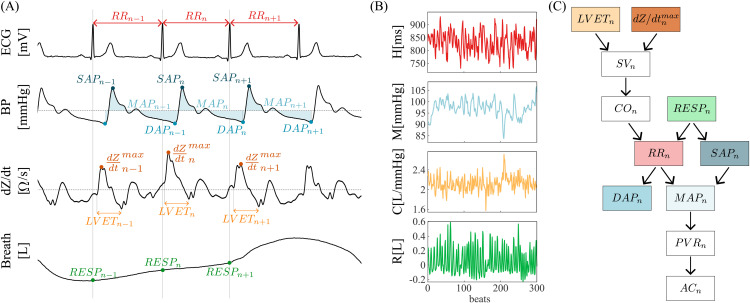
Data acquisition. **(A)** Acquired signals (electrocardiogram, ECG; blood pressure, BP; impedance cardiography, dZ/dt; respiration signal, Breath) and corresponding extracted time series (heart period, RR; mean arterial pressure, MAP; respiration amplitude, RESP). Arterial compliance (AC) was computed exploiting parameters extracted from the dZ/dt and the BP waveforms. **(B)** Exemplary *300*-beat stationary time series of RR (*H*), MAP (*M*), AC (*C*) and RESP (*R*). **(C)** Convention for the choice of zero-lag effects. For visual purposes, colors of the exemplary time series in panel b and the boxes in panel c correspond to the series points depicted in panel a.

Electrocardiogram (ECG, CardioFax ECG-9620, NihonKohden Japan), and arterial blood pressure (BP) curve from finger, with the subsequent brachial arterial BP reconstruction by the photoplethysmographic volume-clamp method (Finometer Pro, FMS Netherlands), were simultaneously and noninvasively recorded (Fig 1A, ECG and BP). A hydrostatic height correction unit was attached on the measured arm at the level of heart to correct for effects of hydrostatic pressure associated with the vertical distance between finger and heart level. Respiration signal (Breath) was measured through the respiratory inductance plethysmography method (RespiTrace, NIMS, Miami Beach, FL, USA) using both thoracic and abdominal impedance belts following qualitative diagnostic and fixed volume calibrations (Fig 1A, Breath). Impedance cardiography (ICG, CardioScreen 2000, Medis, Germany) was also performed, thus enabling continuous beat-to-beat noninvasive monitoring of several indices characterizing myocardial performance and hemodynamics. This method calculates first the changes in the transthoracic impedance (IMP or ΔZ waveform) as a result of the volume and blood flow velocity variations in the aorta, and then the changes in blood volume in the transthoracic region over time, getting the derived IMP waveform as the first mathematical derivative of ΔZ waveform, indicated as dZ/dt (Fig 1A, dZ/dt). All the acquired signals were digitized at a sampling rate of 1 kHz.

### Time series extraction and data pre-processing

Starting from the acquired signals, physiological time series displaying the dynamic activity of cardiovascular and respiratory variabilities were extracted, as detailed in Fig 1A. Heart period (RR) intervals were approximated as the time distance between the $n^{th}$ and the $(n+1{)}^{th}$ R peaks of the ECG (i.e., RR^*n*^). The $n^{th}$ systolic arterial pressure (SAP) value (i.e., SAP_*n*_) was measured as the maximum of the BP signal inside RR_*n*_. The $n^{th}$ diastolic arterial pressure (DAP) value (i.e., DAP_*n*_) was taken as the minimum of BP between the occurrences of SAP_*n*_ and SAP_*n*+1_. Mean arterial pressure (MAP) was calculated as the true integrated mean pressure between the occurrences of DAP_*n*−1_ and DAP_*n*_. The $n^{th}$ respiration amplitude (RESP) value (i.e., RESP_*n*_) was computed sampling the Breath signal on the $n^{th}$ R peak of the ECG.

Cardiovascular measures including stroke volume (SV) and cardiac output (CO) were estimated starting from the dZ/dt waveform. Specifically, the Bernstein and Sramek formula [27] was exploited to compute SV, which is proportional to the maximal systolic ejection speed ($dZ/dt^{max}$) and to the duration of the ejection phase (Left Ventricular Ejection Time, LVET) evaluated in the same beat, while the $n^{th}$ CO value (i.e., CO_*n*_) was computed as the ratio between SV_*n*_ and RR_*n*−1_ [27,28]. Peripheral vascular resistance (PVR) was calculated for each heartbeat (i.e., PVR_*n*_) as the ratio of MAP_*n*_ and CO_*n*_, assuming zero venous pressure at the right atrium. Finally, the value of arterial compliance (AC) was quantified on a beat-to-beat basis through a recently developed method, based on a reliable estimation of the time constant τ, i.e., the rate of the peripheral BP decay during the diastolic phase, as well as on the exploitation of the common relationship between τ, AC and PVR based on the two-element Windkessel model, i.e., $AC_{n}=\tau_{n}/PVR_{n}$ [10]. For any further information on the determination of the AC time series, we refer to [10].

As shown in Fig 1B for a representative subject, stationary segments of 300 consecutive beats were extracted from the original RR, MAP, AC, and RESP time series (henceforth referred to as *H*, *M*, *C*, and *R*, respectively). These windows were selected in REST starting 8 min after the beginning of the measurement, and in HUT 3 min after the position change from supine to tilt, in order to avoid transient changes in cardiovascular parameters. We refer the reader to [11,25,26] for further details about the study protocol, data acquisition and time series extraction.

Classical time domain markers, i.e., the mean and standard deviation of RR ($\mu_{H}\left[ms\right]$, $\sigma_{H}\left[ms\right]$), MAP ($\mu_{M}\left[mmHg\right]$, $\sigma_{M}\left[mmHg\right]$), and AC ($\mu_{C}\left[\frac{L}{mmHg}\right]$, $\sigma_{C}\left[\frac{L}{mmHg}\right]$), as well as the respiratory rate and tidal volume ($f_{R}\left[\frac{1}{min}\right]$, TV[L]), were first computed. Then, time series were pre-processed to remove slow trends through high-pass filtering (zero phase, cut-off frequency 0.0156 Hz) and normalized to zero mean.

### Conditional causality measures

In this work, physiological interactions at rest and in response to orthostatic stress were investigated through the information-theoretic measure of conditional transfer entropy proposed and discussed in [29], with the purpose of identifying and quantifying the direct causal links among the *H*, *M*, *C*, and *R* time series constituting the four nodes of the investigated network. This measure expands the concept of bivariate transfer entropy [30], accounting for the influence of the rest of the network on estimating the strength of causal (i.e., time-asymmetric) coupling between two given nodes. Thus, it resolves whether the interaction between the two considered dynamic random processes is direct or mediated by the other processes.

To give some mathematical background, let us consider a network of *M* dynamic systems, whose activity is described by the set of *M* random processes $\text{X}=\{X_{1},\ldots ,X_{M}\}$. The information-theoretic measure of conditional transfer entropy [29] can be exploited to quantify the direct causal links among the nodes of the network, as it allows the evaluation of the directed information flow from a source process $X_{i}$ to a target process $X_{j}$ (i,j=1,…,M,i≠j) accounting for the effect of the remaining M−2 processes collected in $\text{Z}=\text{X}\backslash \{X_{i},X_{j}\}$. Specifically, considering the random variables describing the present and past states of the processes $X_{i}$, $X_{j}$ and Z  at time *n*, i.e., $X_{j,n}$, $X_{i,n}$, ${\text{Z}}_{n}$, and $X_{j,n}^{-}=\left[X_{j,n-1},X_{j,n-2},\ldots \right]$, $X_{i,n}^{-}=\left[X_{i,n-1},X_{i,n-2},\ldots \right]$, ${\text{Z}}_{n}^{-}=\left[{\text{Z}}_{n-1},{\text{Z}}_{n-2},\ldots \right]$, respectively, the conditional transfer entropy is formulated as [29]:


$$\begin{array}{c} \begin{array}{cc} T_{X_{i}\to X_{j}|\text{Z}} & \;=I(X_{j,n};X_{i,n}^{-}|X_{j,n}^{-},{\text{Z}}_{n}^{-})\\ & \;=𝔼\left[\log\frac{p(x_{j,n}|x_{i,n}^{-},x_{j,n}^{-},{\text{z}}_{n}^{-})}{p(x_{j,n}|x_{j,n}^{-},{\text{z}}_{n}^{-})}\right], \end{array}\; \end{array}$$ (1)

where 𝔼[·] is the expectation operator, p(·|·) the conditional probability, and I(·;·|·) the conditional mutual information.

Specifically, two different formulations of the conditional transfer entropy were applied to physiological data, i.e., the autoregressive model-based (MB) [23,31,32] and model-free (MF) [24,33,34] formulations.

#### Model-based formulation of conditional causality.

Assuming that the oscillatory activity at the network nodes can be described by means of linear Gaussian stationary processes, an autoregressive (AR) model-based definition of conditional transfer entropy can be exploited to describe the causal relationships between the observed data. For Gaussian processes, it was shown that the concept of transfer entropy, formulated as a measure of causal information transfer between joint processes [30], is entirely equivalent to the concept of Wiener-Granger causality [35], which was formalized in terms of linear autoregression by Granger [31]. A straightforward time domain formulation of conditional Granger causality based on a multivariate linear autoregressive model and extended to include zero-lag effects has been proposed in [23] and is used in this study.

The dynamic interactions among the *M* processes in X  can be described by the vector AR model [36]:


$${\text{X}}_{n}=\sum_{k=0}^{p}{𝐀}_{k}{\text{X}}_{n-k}+{\text{U}}_{n},$$ (2)

where *p* is the model order, defining the maximum lag used to quantify interactions, ${\text{X}}_{n}=[X_{1,n},\ldots ,X_{M,n}{]}^{T}$ is a *M*-dimensional vector collecting the present state of all processes, ${\text{X}}_{n-k}={\left[X_{1,n-k},\ldots ,X_{M,n-k}\right]}^{⊺}$ is a *M*-dimensional column vector collecting the past state of all processes at lag *k*, ${𝐀}_{k}$ is the M×M matrix of the model coefficients relating the present with the past of the processes at lag *k*, and ${\text{U}}_{n}=[U_{1,n},\ldots ,U_{M,n}{]}^{T}$ is a *M*-dimensional vector with M×M positive definite covariance matrix Σ, collecting the present state of the zero-mean white noises $\left\{U_{1},\ldots ,U_{M}\right\}$, the latter uncorrelated with ${\text{X}}_{n-k},k=\tau ,\ldots ,p$.

To compute measures of Granger causality from $X_{i}$ to $X_{j}$ conditioned on Z , a restricted linear regression is derived starting from the vector autoregressive representation in (2):


$${\text{X}}_{n}^{i}=\sum_{k=0}^{p}{\tilde{𝐀}}_{k}{\text{X}}_{n-k}^{i}+{\tilde{\text{U}}}_{n}^{i},$$ (3)

where ${\text{X}}_{n}^{i}$ and ${\text{X}}_{n-k}^{i}$ are (M−1)-dimensional vectors collecting the present and past states of all processes in X  except $X_{i}$, i.e., ${\text{X}}^{i}=\{X_{1},\ldots ,X_{i-1},X_{i+1},\ldots ,X_{M}\}$, ${\tilde{𝐀}}_{k}$ is the (M−1)×(M−1) matrix of model coefficients relating the present and past samples of the processes in ${\text{X}}^{i}$, and ${\tilde{\text{U}}}_{n}^{i}$ is a (M−1)-dimensional vector with covariance matrix Λ, collecting the present state of the zero-mean white noises $\left\{{\tilde{U}}_{1},\ldots ,{\tilde{U}}_{i-1},{\tilde{U}}_{i+1},\ldots ,{\tilde{U}}_{M}\right\}$. It is important to note that in both (2) and (3) the lag counter starts from k=0 rather than from k=1 as typically assumed in linear regression models. With this choice, zero-lag interactions are taken into account in the description of the relationship between the present and past states of the *M* processes; the vector autoregressive models (2), (3) are referred to as *extended* [37], from which measures of conditional causality in the time domain can be derived [23].

To compute extended measures of conditional Granger causality from $X_{i}$ to $X_{j}$, unrestricted and restricted linear regressions combining both instantaneous and lagged effects (τ=0) are derived from the vector autoregressive models in (2) and (3):


$$X_{j,n}=\sum_{k=0}^{p}{𝐀}_{j,k}{\text{X}}_{n-k}+U_{j,n},$$ (4)

$$X_{j,n}=\sum_{k=0}^{p}{\tilde{𝐀}}_{j,k}{\text{X}}_{n-k}^{i}+{\tilde{U}}_{j,n},$$ (5)

where ${𝐀}_{j,k}$ and ${\tilde{𝐀}}_{j,k}$ represent the $j^{th}$-row coefficients of ${𝐀}_{k}$ and ${\tilde{𝐀}}_{k}$, respectively, weighting the present and past samples of all processes in X  for the unrestricted regression (4), and the present and past samples of all processes in ${\text{X}}^{i}$ for the restricted regression (5). The residuals of the unrestricted (4) and restricted (5) regressions have variances $\sigma_{jj}^{2}$ and $\lambda_{jj}^{2}$, respectively, and are extracted as the $jj^{th}$ elements of the covariance matrices Σ and Λ, respectively. The extended conditional Granger causality measure is computed by comparing the variance of the residuals of the two models,


$$F_{X_{i}\to X_{j}|\text{Z}}=log(\frac{\lambda_{jj}^{2}}{\sigma_{jj}^{2}}),$$ (6)

and, in the case of Gaussian processes, it corresponds to the transfer entropy $T_{X_{i}\to X_{j}|\text{Z}}$ (1) up to a factor *2*, i.e., $T_{X_{i}\to X_{j}|\text{Z}}=\frac{1}{2}F_{X_{i}\to X_{j}|\text{Z}}$ [32].

As regards estimation of the above measures of causality, model identification was performed using the vector least-squares method [36] and model orders were selected for each subject and condition according to the Akaike Information Criterion.

#### Model-free formulation.

The non-parametric approach used in this work for evaluating direct causal measures is based on the idea that the probability density around a data point is inversely related to the distance from its nearest samples, i.e., on the *k*-nearest neighbour method [33]. Although this approach is widely employed for its reliability and robustness, the accuracy of the estimates is lower when the estimated probability distributions are simply replaced in (1). Indeed, the combination of terms evaluated on spaces of different dimensions causes the presence of significant bias in the estimates. The formulation introduced by Kraskov, Stögbauer and Grassberger limits this bias by using the same range search in all the spaces after defining the searching distance in the highest dimensional space [34].

Exploiting the decomposition of the conditional mutual information in different entropy quantities, the conditional transfer entropy can be estimated as [24]:


$$\begin{array}{c} \begin{array}{cc} T_{X_{i}\to X_{j}|\text{Z}} & \;=H(X_{j,n},V_{j,n},V_{z,n})-H(V_{j,n},V_{z,n})\\ & \;-H(X_{j,n},V_{j,n},V_{i,n},V_{z,n})+H(V_{j,n},V_{i,n},V_{z,n}), \end{array}\; \end{array}$$ (7)

where $V_{j,n}$, $V_{i,n}$ and $V_{z,n}$ are the embedding vectors of dimension $d_{j}$, $d_{i}$ and $d_{z}$, respectively, approximating $X_{j,n}^{-}$, $X_{i,n}^{-}$ and ${\text{Z}}_{n}^{-}$.

Using the maximum norm to calculate distances, the entropy term in the highest dimensional space, i.e., $\left[X_{j,n},V_{n}\right]$ with $V_{n}=\left[V_{j,n},V_{i,n},V_{z,n}\right]$, is estimated as:


$$H(X_{j,n},V_{n})=-\psi (k)+\psi (N)+(d_{j}+d_{i}+d_{z}+1)\langle \ln\epsilon \rangle ,$$ (8)

where ⟨·⟩ is the average operator, ψ(·) the digamma function, *N* the number of observations, and ϵ/2 the distance between the considered sample and its $k^{th}$ neighbour. The entropy terms defined in the projected lower dimensional spaces are then estimated as:


$$\begin{array}{c} \begin{array}{cc} & \;H(V_{n})=\psi (N)-\langle \psi \left(N_{V_{n}}+1\right)\rangle +(d_{j}+d_{i}+d_{z})\langle \ln\epsilon \rangle ,\\ & \;H(X_{j,n},V_{j,n},V_{z,n})=\psi (N)-\langle \psi \left(N_{X_{j}V_{j}V_{z}}+1\right)\rangle \\ & \;\;\;\;\;+(d_{j}+d_{z}+1)\langle \ln\epsilon \rangle ,\\ & \;H(V_{j,n},V_{z,n})=\psi (N)-\langle \psi \left(N_{V_{j}V_{z}}+1\right)\rangle \\ & \;\;\;\;\;+(d_{j}+d_{z})\langle \ln\epsilon \rangle , \end{array}\; \end{array}$$ (9)

where $N_{V_{n}}$, $N_{X_{j}V_{j}V_{z}}$ and $N_{V_{j}V_{z}}$ are the number of points at distances smaller than ϵ/2 from the considered sample in the spaces $V_{n}$, $\left[X_{j,n}V_{j,n}V_{z,n}\right]$ and $\left[V_{j,n}V_{z,n}\right]$, respectively. Substituting the entropy terms (8) and (9) in (7), the nearest-neighbour estimate of the conditional transfer entropy is obtained as:


$$\begin{array}{c} \begin{array}{cc} T_{X_{i}\to X_{j}|\text{Z}} & \;=\psi (k)+\langle \psi \left(N_{V_{j}V_{z}}+1\right)-\psi \left(N_{X_{j}V_{j}V_{z}}+1\right)\\ & \;-\psi \left(N_{V_{n}}+1\right)\rangle . \end{array}\; \end{array}$$ (10)

Finding embedding vectors that closely approximate the infinite-dimensional past states of the processes is a critical step in estimating theoretical-information measures using MF approaches. When working with data of finite length, e.g., the 300 samples typically used for the analysis of short-term physiological time series, the employment of high-dimensional detailed vectors to provide a more complete description of past processes leads to the curse of dimensionality and unreliable estimates of entropy quantities due to the estimator sensitivity to time series and signal patterns length [38]. An alternative selection technique to the uniform embedding approach, which simply uses a fixed number of equally spaced samples, was introduced to limit the size of the descriptive patterns by selecting the most informative time-lagged samples in evaluating the information exchanged across the interacting processes. Specifically, the non-uniform embedding approach described in [24] was exploited in this work.

Considering a set of candidates including all possible states of the processes up to a maximum lag *L*, i.e., $\Omega =\Omega^{X_{i}}\cup \Omega^{X_{j}}\cup \Omega^{\text{Z}}$ being $\Omega^{X_{i}}=\{X_{i,n-\tau }\ldots X_{i,n-L}\}$, $\Omega^{X_{j}}=\{X_{j,n-1}\ldots X_{j,n-L}\}$ and $\Omega^{\text{Z}}=\{{\text{Z}}_{n-\tau }\ldots {\text{Z}}_{n-L}\}$ with τ the initial lag (τ=0 when instantaneous effects are taken into account and τ=1 when they are not; see next subsection for details about instantaneous effects), an iterative approach is applied to select the components of $V_{n}$ which maximize the information shared by the target variable $X_{j,n}$ and the candidate variables. Starting with an empty embedding vector $V_{n}^{0}$, for steps d≥1, the candidates ${\hat{W}}_{n}$ are selected from a subset $W_{n}\in \Omega \backslash V_{n}^{d-1}$ as:


$${\hat{W}}_{n}=\arg\;\max_{W_{n}}I(X_{j,n};W_{n}|V_{n}^{d-1}).$$ (11)

After each step, a surrogate-based approach is used to test the significance of the information brought to the target variable by the selected candidates. Specifically, the conditional mutual information $I(X_{j,n};{\hat{W}}_{n}|V_{n}^{d-1})$ is compared with a threshold obtained as the $100(1-\alpha {)}^{th}$ percentile of a distribution resulted computing the same measure over $N_{s}$ realizations of the process obtained shuffling randomly and independently the samples of ${\hat{W}}_{n}$ and $X_{j,n}$. Thus, the selected candidate is added to the embedding vector if $I(X_{j,n};{\hat{W}}_{n}|V_{n}^{d-1}$ is higher than the threshold, resulting in $V_{n}^{d}=\left[V_{n}^{d-1}{\hat{W}}_{n}\right]$.

In this work, after normalizing time series to unit variance, the MF causality measures were estimated by fixing a maximum lag of *10* samples and setting a number of neighbors k=10. Moreover, $N_{s}=100$ realizations of surrogate data were generated to apply the non-uniform embedding technique and the significance threshold was fixed to α=0.05.

#### Instantaneous effects among physiological time series.

The assessment of causality, intended as the influence that a driver process exerts on a given target, requires a proper handling of instantaneous (zero-lag) effects. In practical physiological time series analysis, instantaneous causality shows up whenever the time resolution of the measurements is lower than the time scale of the lagged causal influences occurring among the analysed processes. This non-delayed effect can arise due to non-physiological factors (e.g., unobserved confounders) or fast (i.e., in the analysis of physiological time series, within-beat) physiologically meaningful interactions [37]. The importance of considering instantaneous effects in the analysis of cardiovascular interactions, where zero-lag interdependencies are expected to contribute significantly to the baroreflex mechanism [37], and of cardiorespiratory interactions, where the information transfer from respiration to heart rate is expected to occur within the cardiac beat [39], was previously documented [23,37].

In this work, in addition to lagged interactions, instantaneous effects were accounted for and suitably determined according to the measurement convention displayed in Fig 1A. This was done by visually inspecting the temporal sequence of the investigated physiological indices or the parameters directly involved in their estimation, as well as by considering that the cause always precedes its effect in time. Accordingly, the zero-lag effects were set along the directions depicted in Fig 1C, i.e., $RESP_{n}\to RR_{n}$, $RESP_{n}\to MAP_{n}$, $RESP_{n}\to AC_{n}$, $RR_{n}\to MAP_{n}$, $RR_{n}\to AC_{n}$, and $MAP_{n}\to AC_{n}$. These zero-lag interactions were considered in both the model-based and model-free [24] estimators described in the previous subsections. Specifically, the model used for parametric estimation incorporates these instantaneous effects into the zero-lag connectivity matrices ${\text{A}}_{0}$ and ${\tilde{𝐀}}_{0}$ of Eqs (2) and (3), while all other entries of these matrices are zero [23], and the model-free procedure included these effects together with the time lagged ones in the set of candidates possibly affecting the target variable [24]. Notably, the chosen instantaneous effects create a directed acyclic graph (DAG) which avoids self-loops of zero-lag effects, which would make the extended model (2) unidentifiable. Moreover, the sequential ordering of instantaneous effects within the DAG (in this case, $RESP_{n}\to RR_{n}\to MAP_{n}\to AC_{n}$) prevents from conditioning on collider variables that would introduce spurious associations (e.g., $MAP_{n}$ is not included in **Z** when the target is $X_{j}=RESP_{n}$ because $MAP_{n}\to RESP_{n}$ is not a plausible instantaneous effect, while it is included in **Z** when the target is $X_{j}=AC_{n}$ because $MAP_{n}\to AC_{n}$ is a physiologically plausible instantaneous effect).

### Surrogate and statistical data analysis

To statistically confirm the presence of the given interconnection between assessed variables, we performed surrogate data analysis where measured coupling strength values obtained by either MB or MF approaches were compared with the values for the artificially prepared groups of signals. Preparation of these surrogate signals preserved properties of the individual signals but destroyed interconnections among them. Specifically, the statistical significance of the estimated causality measures referred to individual links was assessed for each single subject in both REST and HUT conditions. One-hundred surrogate time series were generated (i) through the *iterative Amplitude Adjusted Fourier Transform* procedure [40] as regards the MB indices, and (ii) by randomly shifting the target over time (minimum shift of 20 lags) and leaving all the other series unchanged as regards the MF indices. The MB or MF causality measure computed on the original time series was deemed as statistically significant if its value was higher than the 95_*th*_ percentile of the distribution derived by computing the same measure on the set of surrogates. In this work, the degree of significance *s* for a given MB or MF conditional causality measure is intended as the ratio between the number of subjects for which the measure was deemed as statistically significant over the whole cohort and was subdivided into three classes, namely s=[25−50]% (low significance), s=[50−75]% (medium significance) and s=[75−100]% (high significance). The first of these classes was arbitrarily intended as not statistically relevant.

Given the small size of the surveyed population, non-parametric statistical tests were applied to assess statistical differences between physiological indices evaluated in the two phases of the experimental protocol, i.e., REST and HUT conditions. Specifically, the Wilcoxon signed rank test for paired data was applied on classical time domain markers, i.e., mean and standard deviation for *H*, *M*, and *C* time series, and respiratory rate and tidal volume for *R* time series. Moreover, the Wilcoxon signed rank test for paired data was also applied on conditional causality measures evaluated for each pair of nodes using both MB and MF approaches by using the Benjamini-Hochberg procedure to evaluate the false discovery rate (FDR) as to prevent error I type due to multiple comparison. For all the statistical tests, the significance level was set to 0.05.

## Results

### Classical time domain markers of physiological time series

**Fig 2:**
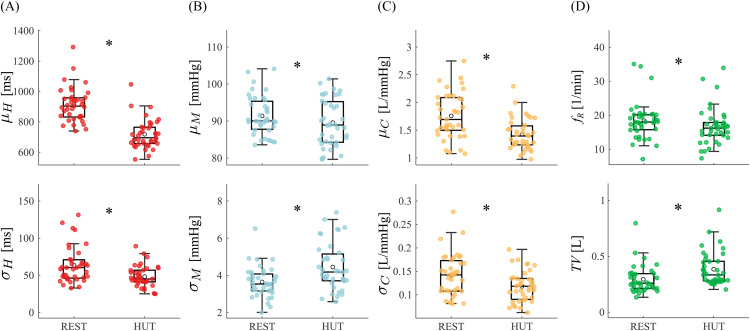
Series markers. Mean (top row) and standard deviation (bottom row) of the **(A)** RR (*H*), **(B)** MAP (*M*) and **(C)** AC (*C*) time series depicted as boxplot distributions and individual values in the supine (REST) and orthostatic (HUT) positions; **(D)** respiratory rate (top row) and tidal volume (bottom row) computed on the Breath signal. Wilcoxon signed rank test for paired data: REST vs. HUT, (*) p<0.05.

Fig 2 shows the results of the analysis performed on classical markers of mean and standard deviation of cardiovascular variability time series, and on respiratory rate and tidal volume as regards the breathing signal. We observe a significant decrease of the mean RR and AC, as well as of their variabilities (panels a and c). On the other hand, the significant decrease of MAP is accompanied by a significant increase of its variance (panel b). As regards respiratory indices, a significant decrease of the respiratory rates and an increase of the tidal volumes are found with HUT (panel d).

### Model-based vs. model-free approaches

**Fig 3:**
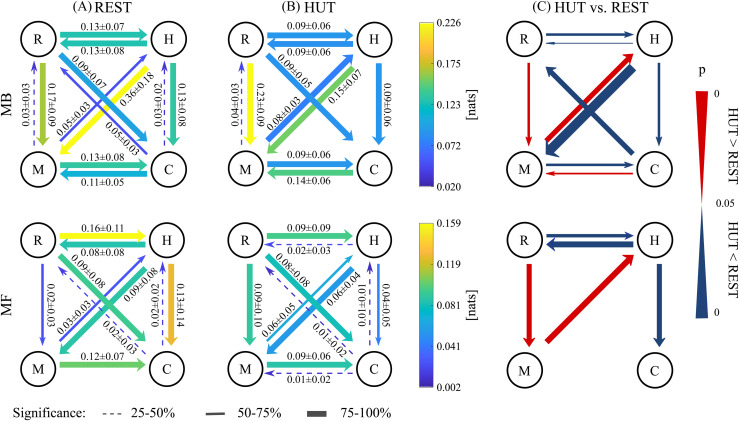
Causal networks. Directional networks including RR (*H*), MAP (*M*), AC (*C*) and RESP (*R*) dynamics computed from model-based (MB) (top) and model-free (MF) (bottom) conditional causality measures in the **(A)** REST and **(B)** HUT conditions. The average across subjects is reported in color and expressed in numerical form together with the standard deviation (mean ± std. dev.), while the width of the arrows displays results from surrogate data analysis. **(C)** Directional networks indicating the significant differences between conditional causality measures computed in the REST and HUT conditions. Wilcoxon signed rank test for paired data (*p* < 0.05) corrected with FDR controlling procedure: red, HUT > REST and blue, HUT < REST. Progressive change of the width of the arrows indicates increase/decrease of *p*-values in logarithmic scale. Arrows are shown only if the corresponding percentage of significance is above 50% for at least one of the conditions.

In this section, we display results relevant to Fig 3A, 3B, with the aim to compare MB (top row) and MF (bottom row) approaches considering the statistical significance of the interactions in the investigated physiological network. The strength of the causal couplings within the network is quantified in natural units ([nats]) of information.

Cardiorespiratory dynamics are strongly detected by both MB and MF approaches during REST and HUT states. Importantly, the causal interaction R→H strongly prevails over the opposite direction in REST if these dynamics are investigated using MF causality measures (Fig 3A, bottom). As regards cardiovascular interactions, i.e., the well-known relationship between MAP and RR, the baroreflex direction (M→H) is significantly captured in both conditions and by both approaches, with s>75% during HUT using the MB method (Fig 3B, top) and 50%<s<75% in all the other cases. The opposite link (the feedforward direction, H→M) represents a stronger mechanism captured by both MB and MF approaches with s>75%. The causal effect of MAP on AC (M→C) is always captured by both MB and MF approaches (s>75%). The opposite interaction C→M is significantly detected only by the MB approach (s>75%, Fig 3A, 3B, top). The causal interaction directed from the heart rate to large vessels (H→C) is disclosed by both approaches with high significance, except for the MF measure in the HUT state (50%<s<75%, Fig 3B, bottom). The causal effect of respiratory variability on compliance of arteries (R→C) is always detected with high significance by both approaches (s>75%); the opposite influence of compliance on respiration seems to have an important role only during the supine resting state if investigated with the MB approach (50%<s<75%, Fig 3A, top), even though absolute values are very low. The causal interaction R→M is always observed with high significance (s>75%) in HUT using both MB and MF causality measures (Fig 3B), and at REST using the MB approach only (Fig 3A, top).

### Comparison between the supine resting state and the head-up tilt experimental conditions

In this section, we display results relevant to Fig 3C, with the aim to compare the REST and HUT experimental conditions.

Cardiorespiratory interactions are strongly influenced by postural changes. The causal interaction H→R decreases significantly with HUT, and this diminished effect is visible exploiting both MB and MF approaches; the dynamics in the opposite direction are also weaker during postural stress, with slightly higher statistical importance by employing the MF approach (Fig 3C, bottom). The baroreflex effect of MAP on RR increases significantly with HUT; the augmentation is captured by both methods. Conversely, the MB causality measure significantly decreases along the feedforward in HUT with a very low *p*-value (Fig 3C, top). The MB method is also able to detect an increase of the causal interaction C→M, and a decrease of M→C (Fig 3C, top). Respiratory-related changes of MAP are enhanced in HUT, with R→M increasing using both approaches. The MB approach also identifies a decreased influence of the arterial compliance dynamics on the ventilatory activity (Fig 3C, top). Finally, the causal effect of RR on AC decreases significantly during HUT for both approaches, with higher statistical importance by using the MF method (Fig 3C, bottom).

## Discussion

This work points out the complex interplay between the dynamical activities of cardiovascular variables in different experimental conditions, by considering the causal dependencies among pairs of time series within a dense and intricate network of interactions. In particular, the combination of model-based and model-free approaches allowed us to investigate both the linear and nonlinear characters of these coupled interactions. From a physiological point of view, our findings reveal putative nonlinear mechanisms of interaction at the basis of some causal links within the analyzed network, providing further insights on the interplay between physiological variables during the supine resting state and the orthostatic challenge.

### Methodological focus

Autoregressive model-based and model-free approaches have been used to describe the interplay among the multiple nodes of complex physiological networks in different states. The comprehension of their advantages and disadvantages, as well as of their methodological differences, is crucial for understanding and interpreting these coupled interactions.

Theoretical definitions of transfer entropy, based on the concept of Shannon entropy [41], involve the use of variables of infinite dimensionality that sample the entire temporal evolution of the processes [42]. In practice, realizations of these variables are of finite length; their handling is simpler when working with linear model-based approaches. However, constraining the analysis on a specific model structure can limit the ability to detect and describe nonlinear interactions and complex behaviors. Model-free approaches are then often necessary to overcome the drawbacks of losing generality and inability to identify potential nonlinear dynamics. On the other hand, they require direct, non-parametric estimation of probability distributions, which makes them computationally demanding.

In Fig 3A, 3B, the strength and statistical relevance of the causal interactions within the network are represented by different colors and widths of the arrows, respectively. The investigation of these two indicators, assessed via MB or MF approaches, might reflect prevalence of linear or nonlinear mechanisms beyond the observed phenomena. Indeed, although the absolute values of the computed MB and MF measures differ and are not directly comparable, as MF estimators are known to underestimate entropy measures and causality [24,43], it is still important to correlate the networks obtained using the two approaches in terms of presence/absence of specific links and their statistical weight. Nevertheless, it is necessary to account for the discrepancies between the two procedures in terms of estimation bias and variance, which could affect the obtained results. While the absence of a significant link in the MB approach, that is significant using the MF, would indicate the presence of nonlinear dynamics governing that physiological direction, it is expected that all links assessed as significant through the MB method would also be deemed significant with the MF estimator, since it is able to discover linear dynamics. As evidenced in Fig 3, the latter is valid for nearly all the examined links, with the exception of the causal directions from M to R, and from C to M. Accordingly, in order to interpret the difference across the two inferred networks, it is also necessary to consider the effect of the estimation variance, particularly affecting the MF estimation approach. In this regard, the high variance of the causality measures obtained on surrogate data, especially for weaker causal dynamics, results in an assessment of that link as not significant, in contrast to the MB approach.

### The network physiology behind the resting state

The study of resting state cardiovascular networks is of great interest for research and clinical applications. Physiological parameters fluctuate in order to maintain body homeostasis, reflecting the ability of healthy subjects to respond appropriately to physiological changes [44]. In this section, we interpret results relevant to the supine resting state by describing the mechanisms underlying the beat-to-beat variabilities of heart rate, blood pressure and arterial compliance within the investigated network of interactions.

#### Heart rate variability.

It is well known that heart rate is one of the physiological parameters characterized by the highest variability. Heart rate variability (HRV) varies with age and gender [44], and its lack or depression have been described as indicators of several pathological states, e.g., nervous system disorders [45], diabetes [46], arterial hypertension [47], and myocardial infarction [48]. The physiology behind the regulation of cardiac dynamics is complex, but most studies agree that the main components of the short-term normal sinus rhythm variability are related to the control exerted by the autonomic nervous system (ANS) [44,49]. During ventilation, the activity of the sinoatrial node is directly influenced by the modulation of vagal neurons directed to the heart, controlled by the central respiratory drives (direct communication between respiratory and cardiomotor centers), the lung inflation reflex, and the changes in arterial blood pressure transferred to heart rate via baroreflex [44,50,51]. These mechanisms result in the so-called respiratory sinus arrhytmia (RSA), for which there is an increase of heart rate during the inspiration phase and a decrease during the expiration phase of ventilation [49,52]. While the physiological causes and effects of the RSA mechanism have been widely studied and discussed throughout the past decades [18,52,53], very little is known about the cardiac-driven respiratory variability. It is widely believed that the coupling between the cardiovascular and respiratory systems is unidirectional, i.e., the respiratory rhythm influences the heart rate via vagal nerve traffic oscillations and, to a much lower extent, via direct mechanical influence on the sinoatrial node [54]. Nevertheless, some evidence conflicts with this theory suggesting that the respiratory oscillator in the central nervous system is not always dominant, i.e., both the influence of respiration on heartbeat (i.e., the RSA mechanism) and the influence of heartbeat on respiration are important for cardiorespiratory synchronization mechanisms [55]. Among them, cardioventilatory coupling is the entrainment phenomenon in which ventilation and heartbeats become synchronized in whole number ratios and heartbeats fall in constant timing relationship with the onset of inspiration (see, e.g., [6,56]). The mechanism has been suggested to be likely caused by either a cardiac-induced onset of inspiration through an unknown haemodynamic afferent pathway [56], or the effects of baroreceptor reflexes and ANS activity [6]. An additional phenomenon that is known to characterize mutual relations between cardiac and ventilatory activity is the cardio-respiratory phase synchronization (CRPS). In contrast to the RSA mechanism, which involves the periodic modulation of heart rate amplitude within each respiratory cycle, CRPS leads to an alignment of the ECG R-peaks at specific phases of the respiratory cycle, thereby inducing phase synchronization phenomena [57,58].

Our results confirm the presence of strong closed-loop interactions involving cardiorespiratory dynamics. Indeed, both the MB and MF estimators were able to detect significantly relevant causal interactions along the directions R→H and H→R (Fig 3A), suggesting that the mechanisms involved in the regulation of cardiorespiratory dynamics are significantly strong and bidirectional. Remarkably, some studies documented the importance of using nonlinear approaches, either based on entropy measures or nonlinear autoregressive models, to investigate the complex interactions between respiration and heart rate [7,39]. Indeed, it was demonstrated that the interplay between the cardiac and respiratory systems may lead to the rise of nonlinear RSA dynamics [7], herein well identified by the MF estimator (Fig 3A, bottom). Moreover, as also highlighted in [59], cardiorespiratory patterns of interaction are characterized by a relevant instantaneous influence of the breathing dynamics on the cardiac activity. In our work, the zero-lag sample of the RESP series was selected for more than 50% of subjects by using the MF estimator, thus confirming the major role played by respiratory-driven instantaneous interactions directed to the heart.

It is worth noting that heart rate is influenced not only by respiration but also by MAP to a lower extent (Fig 3A). The baroreflex interaction was found to be significant in most of subjects, thus confirming the major role played by arterial pressure in governing beat-to-beat changes of heart rate and vascular tone [50,60].

#### Blood pressure variability.

In the supine resting state, together with the heart rate, arterial blood pressure also exhibits spontaneous fluctuations and is strongly influenced by ventilatory and sympathetic activities [61]. Although the study of physiological mechanisms involving BP variability (BPV) is usually assessed by SAP or DAP measurements, the use of mean arterial pressure has recently gained support [28]. It was found that, especially at rest, the mean arterial pressure is most tightly coupled with RR [28], thus opening the possibility to exploit this input signal instead of systolic BP for baroreflex analysis. Starting from this frontier, in this work we investigated MAP-based cardiovascular and vascular-respiratory interactions.

With regard to the influence exerted by respiration on BP, the significant causal interaction from RESP to MAP (Fig 3A) is likely due to the cyclic intrathoracic pressure changes that occur during ventilation and lead to alterations in venous return and fluctuations in BP values [62], independently of heart rate changes inducing cardiac filling fluctuations. Overall, our results indicate that the respiratory-driven variability of MAP during the resting state may show a linear character, due to both the observed higher values and significance of the causal coupling R→M assessed through the MB approach compared to the MF approach.

Besides, the resting-state BPV is known to be strongly influenced by a relevant non-baroreflex effect (commonly known as feedforward) led by changes in heart rate, reasonably due to the Windkessel [63] and/or Frank–Starling [64] mechanisms. In this regard, some studies documented a predominance of the cardiovascular effects of heart rate on diastolic BP (run-off effect interaction), as well as of diastolic on systolic BP, which together constitute the pathway heart rate - diastolic BP - systolic BP explaining the high amount of information flowing from RR to SAP in the supine resting state [25,65]. Despite these mechanisms have not been investigated nor analysed in terms of the MAP time series yet, the strong dependence of MAP on DAP and SAP values (with MAP being computed from the integral of the BP curve between two consecutive DAP values) makes the MAP series interesting from this perspective as well. Indeed, our results confirm a strong feedforward mechanism and thus highlight the major role played by MAP within this chain of interactions (Fig 3A). Noteworthy, the selection of a percentage of zero-lag samples by the MF method slightly lower than 50% across subjects, as well as the highly significant causal interaction H→M detected through the MB (Fig 3A, top), suggest that the instantaneous effect directed from heart period to mean arterial pressure may have an important role in characterizing cardiovascular dynamics.

Causal effects on BP can also be ascribed to arterial compliance variability. The interaction directed from AC to MAP appears to be significantly relevant in the case of causality measures computed with the MB approach (Fig 3A, top). Although these dynamics seem to be more evident in pathological conditions or with increasing age [66,67], the beat-to-beat modulation of systolic BP due to arterial compliance variability may play an important role even in youth and healthy physiological conditions. As suggested by previous studies, this can be explained by considering the potential effects of the SV modulations (thus of the compliance) on the arterial system (thus on BP variability) conveyed by respiration [68] and venous blood pool control [69]. Moreover, previous studies documented an important role of PVR as a source of MAP changes [70], although the causal interaction PVR→BP has been investigated only with bivariate approaches (see, e.g., [26]). It has been suggested that PVR, determined by vasomotion in smaller arteries, influences BP by diastolic blood pressure decay (short-term changes) and changes in venous return associated with blood redistribution (long-term changes). Since AC is herein computed from τ and PVR, we can argue that the influence of AC on MAP can be due to the direct effect of PVR on BP.

#### Arterial compliance variability.

With regard to the variability of arterial compliance, the investigation of this parameter has emerged recently in the literature. Recent studies have observed how its regulatory mechanisms are complex and not clearly understood yet [10,11,13]. Remarkably, being a non negligible physiological factor strongly influencing AC, the direct action of sympathetic nerves on the vascular tone of large vessels may play the role of an unobserved confounder when interpreting the coupled interactions from and to AC [71].

As supported by previous studies [12], blood pressure plays an important role in the modulation of pulse wave velocity (PWV) in the supine rest. Assuming PWV as a surrogate index of compliance [10], this is likely reflected by a significant causal interaction along the direction M→C, revealed by both MB and MF approaches (Fig 3A). It is worth noting that the relationship between mean arterial pressure and compliance can be affected by several factors. Indeed, both the weight of the zero-lag effect of MAP on AC, which was selected in more than 50% of subjects by the MF estimator, and the sympathetic activity of the ANS, which can be considered as a common driver for BP and AC, may have an influence on the strength and the significance of the coupling directed from mean arterial pressure to vessel compliance. Additionally, the sympathetic-driven effect of cardiac contractility along the cardiac inotropic arm of the baroreflex should be also taken into account [26], since beat-to-beat modifications of BP at rest may have an impact on the relationship between BP and AC.

Our results also reveal the influence of both heart period and breathing patterns on arterial compliance at rest (Fig 3A). Several studies have demonstrated a significant effect of heart rate fluctuations on compliance variability independently of autonomic control and BPV [12,67,72]. An increase in heart rate has been associated to modifications of the cardiac cycle and a reduction in the time available for recoil, resulting in vascular stiffening and arterial compliance decrease, due to the viscous and inertial components of the arterial walls [67,73]. The relationship between heart rate and vascular compliance has been investigated indirectly in the most of previous works on the topic. Indeed, assuming PWV as a surrogate for AC [10], some studies have shown that PWV is indirectly related to RR independently of BP [12,67,72], supporting the hypothesis that the effect of heart period on arterial compliance could show nonlinear features in the supine resting state. Despite the sympathetic activity of the ANS has an effect directed to the heart which cannot be neglected and may play a common drive role here, our results show a significant strong causal interaction from RR to AC which cannot be ascribed to the confounding effects of respiration nor BP variability (Fig 3A). Furthermore, we found that the zero-lag effect from RR to AC is selected in more that 70% of subjects in the resting state using the MF approach, demonstrating the importance of embedding instantaneous effects into the analysis.

As regards the influence exerted by respiratory variability on compliance, it is known that small changes of intrathoracic pressure and lung volume during spontaneous ventilation can independently affect arterial compliance through changes in atrial filling or preload and modifications in transmural pressure exerted on the arterial wall [74]. This can determine modifications of some cardiovascular parameters, such as SV, as well as of the mechanical properties of the arterial wall [75,76]. Looking at our results, the causal interaction R→C was detected with the highest significance by both approaches (Fig 3A). Interestingly, despite respiratory activity may have an indirect influence on compliance through modulation of heart period and BP, herein we get around the issue by utilizing conditional causality measures and separating the link from confounding external effects. This confirms that a direct effect of respiratory variability on vessel compliance is likely to occur in the supine resting state and may reflect the effect of cyclic changes in transmural pressure on arterial wall stiffness. Moreover, the high significance of this causal link in both time domain MB and MF measures can be ascribed to a great influence of the zero-lag effect, which indeed was selected for more than 50% of subjects by the MF estimator.

### Modification of network interdependencies in response to the orthostatic stress

In this section, we focus on how the causal relationships explored in the supine resting state are adjusted in response to the orthostatic challenge, in order to better understand the complex physiology behind the tilt-induced modification of the interactions among the investigated variables. The orthostatic challenge is known to alter important physiological mechanisms operating in the resting state condition. Orthostasis has been associated to venous pooling of the blood in the lower portion of the body, thus decreasing cardiac filling, cardiac output and stroke volume [77]; in turn, this determines a drop of arterial blood pressure sensed by baroreceptors, vagal inhibition and sympathetic activation directed to the heart and vessels [14,15]. As previously observed in literature [17,26], these mechanisms lead to a reduction of HRV and to an increase of MAP variability (Fig 2A, 2B, bottom).

We observe that the tilt-induced decrease of mean AC (Fig 2C, top) is associated with a decrease of the average inter-beat interval (Fig 2A, top), confirmed in the literature [12], as well as with a decrease of MAP (Fig 2B, top). Assuming that the mean and systolic BP behave similarly in response to the orthostatic challenge [28], our results are in accordance with a previous study [11], where a shift of the SAP-AC curve towards lower values of compliance and systolic BP during HUT was detected. The observation of decreased AC, together with the well-known shift of the SAP-AC curve, reflects a change in the arterial tree properties occurring with orthostasis and thus has been associated with vasomotor activity [11,78]. Indeed, the diminished values of AC during HUT may be attributed to the tilt-induced sympathetic activation, leading to smooth muscle cells constriction in elastic arteries. Contrarily, the decrease of arterial compliance variability is a novel observation (Fig 2C, bottom). Since we are not aware of any relevant citation for this phenomenon, we can speculate about the potential underlying mechanisms: (i) decreased sympathetic control oscillations directed to elastic vessels, even though in contrast to increased oscillations in sympathetic control to resistance vessels (mostly arterioles); (ii) influence of decreased HRV (Fig 2A, bottom), and (iii) lower influence of MAP and RR on stiffer arteries during sympathetic activation.

#### Cardiorespiratory patterns during head-up tilt.

As depicted in Fig 2D and confirmed in previous studies [79,80], the tilt-induced decreased respiratory rates and increased tidal volumes, together with the the well-known parasympathetic activity withdrawal during HUT, leads to a significant RSA weakening, here identified by both MB and MF approaches (Fig 3). This is confirmed by previous studies [81,82] and has been associated to a dominance of the direct central mechanism mediating RSA (i.e., direct communication between respiratory and cardiomotor centers) at supine rest and an increased indirect peripheral mechanism (i.e., ventilation-associated BP changes transferred to heart rate via baroreflex) during orthostasis [18,50,51]. The latter is clearly reflected in our results by the tilt-induced augmented causal interactions R→M and M→H (Fig 3C), which together constitute the indirect peripheral mechanism controlling RSA.

A reduction of the cardiorespiratory coupling is also detected by looking at the causal direction H→R and more visible with the MF approach (Fig 3C, bottom), as also visible by the highest variation in the number of significant measures found with the MF approach (Fig 3A, 3B, bottom). Nevertheless, very little is known about the cardiac-driven respiratory variability and its modification with tilt, as already pointed out in Sect 14. We learn from literature that, although this coupling is poorly seen in alert and active humans, it was clearly observed during relaxation, sleep, anaesthesia and, generally, in conditions of low cognitive and behavioural activity [56]. Here, the significant decrease of the causal coupling along the direction H→R may be associated to the gradually decrease of the complex cardiorespiratory interactions during situations of sympathetic activation [50].

#### Involvement of blood pressure variability in tilt-induced physiological responses.

The increased influence of breathing activity on mean arterial pressure variability, revealed by both MF and MB approaches (Fig 3C), is representative of ventilation-associated BP changes occurring with tilt and due to alterations in intrathoracic pressure, instantaneous lung volume, venous return to the heart, and cardiac output [83]. Previous studies [8,84] suggested that respiratory-related fluctuations of BP can be ascribed to the mechanical thoracic coupling between respiration and vasculature, as well as to the effects of respiratory induced fluctuations of RR. While RSA is thought to buffer respiratory-related BP oscillations during the supine rest [8,84], the mechanical influences of respiration on arterial pressure are thought to be greater in the upright than the supine position. We attempt to confirm this hypothesis: since the causal effect of respiration on MAP is conditioned on the knowledge of all the other variables in the network, the observed increase of the causal coupling R→M is solely due to the enhancement of the mechanical influences of respiration on arterial pressure.

In the complex regulation of BP variability during postural stress, arterial compliance is also involved. Specifically, we found a significant increase of the causal interaction C→M by using the MB approach (Fig 3C, top). As far as we know, the present study represents the first attempt to investigate the causal interactions within a network of four physiological processes comprising arterial compliance. Previous works on the topic suggested that an effect of vascular compliance on arterial pressure may be related to the slow rhythms conveyed to the arterial system through SV modulations; the latter are likely to depend on respiration [68] and on venous blood pool control [69]. Although these mechanisms have not been observed during conditions of postural stress yet, our findings suggest that a relevant portion of the tilt-induced changes of mean arterial pressure should be ascribed to the short-term arterial compliance variability.

The relationship between blood pressure and compliance may have effects on the bidirectional interactions between heart period and BP. Indeed, it has been shown how a transient change in carotid arterial compliance is responsible for a sudden change in the cardiac baroreflex response [71]. Moreover, several works argue that the tilt-induced significantly higher awareness of baroreflex control, due to blood redistribution and baroreceptor unloading during orthostasis, can be also related to the confounding effect of respiration [17,25,65]. In this study, since any external driver of BP variability, i.e., the compliance and respiration variability, is ruled out by the conditioning procedure, we suggest that the observed enhancement of the baroreflex control with the importance of the gravitational stimulus (Fig 3C) is only due to the closed-loop interplay between heart period and arterial pressure, thus confirming previous results obtained in older studies on the topic [25,26,50]. On the other hand, the feedforward direction of the cardiovascular closed-loop, i.e., H→M, is affected by the postural stress, as detected by the MB approach (Fig 3C, top). The highly statistically relevant decrease of this causal coupling is not associated with a correspondent diminished number of significant measures detected with surrogate data analysis (Fig 3A, 3B, top), meaning that the strength of the interaction remains considerably noteworthy within the network. This result is in line with previous studies [25], where the effect of HUT was found to be prominent for the directions RR→DAP and DAP→SAP. The authors suggested that these results can reflect the involvement of other mechanisms influencing DAP and/or contributing to the strength of systolic contraction, such as changes in the PVR associated with tilt-induced sympathetic activation and sympathetic nervous system influence on cardiac contractility, respectively. In our study, we cannot rule out the role exerted by the sympathetic branch of the ANS, although it may have significant effects on the strength of the feedforward interaction and act as a confounder for this link.

All these findings support the idea that, during tilt, changes of BP variability are related to the increase of sympathetic activity induced by the postural stress [65], as well as to the decreased buffering of BP mediated by HRV.

#### Regulation of arterial compliance variability during postural stress.

Here, we speculate about the mechanisms involved in the beat-to-beat regulation of arterial compliance during head-up tilt. Interestingly, we observe a significant decrease of the causal interaction M→C using the MB approach (Fig 3C, top), thus suggesting that orthostasis affects the strength of this link probably due to stiffer vessels. This is a novel finding, since a thorough inspection of this interplay, which is not a straightforward task, is still lacking in the literature. Similar investigations have been carried out in the past years, where the relationship between systolic BP and compliance was found to hold for a given phase but shift during the HUT phase towards lower values of systolic BP and compliance [11,85]. However, the present work goes beyond the classical bivariate analysis, with the aim of ruling out the confounding effects of cardiac and respiratory variables. Following this rationale, we speculate that the decrease of $\mu_{C}$ in HUT (Fig 2C, top) can be partly MAP-dependent, i.e., due to a diminished transfer of information from blood pressure. Nevertheless, it has been suggested that it could depend also on other mechanisms such as sympathetic nervous system activation and/or changes in PVR during HUT [78]. Moreover, although the MF approach did not detect this decrease, it included zero-lag samples of MAP for about 50% of subjects along this direction, thus highlighting the weigh of the instantaneous interaction when taking into account the relationship between arterial pressure and compliance.

A decreased coupling along the direction H→C is also observed with both MB and MF approaches (Fig 3C), as confirmed by surrogate data analysis especially with the MF approach (Fig 3A, 3B, bottom). This finding can be related to the important influence of zero-lag causality (more than half subjects if investigated through the MF approach) on the observed interaction, which could reflect pure mechanical visco-elastic effect. Again, these considerations are absolutely novel and need further studies on the topic to provide deeper physiological reasoning beyond the observed phenomena.

In addition, arterial compliance may play an important role in driving respiratory variability during postural stress, as suggested by the decrease of the causal coupling C→R detected with the MB approach (Fig 3, top). We argue that these dynamics exhibit predominantly linear characteristics and speculate that they may be related to the mechanical interaction between the arterial system and ventilation.

### Limitations and future studies

Several caveats affecting the physiological interpretation of our results can be drawn from this study.

First, it is well known that changes affecting the variability of driver processes can have significant repercussions on the computed causality measures. Tilt-induced diminished variabilities, such as in the case of HRV, may be partly responsible for the observed decrease of some causal interactions, as happens for the causal links H→R, H→M or H→C (Fig 3C). This effect cannot be ruled out but it should be taken into account when translating the obtained results into physiological considerations.

Moreover, we made an effort to build a network characterized by time series at the nodes whose coupled interactions are known to have well-established and significant physiological meanings, i.e., heart period, arterial pressure, and respiration. In this regard, the novelty lies in the possibility to include the beat-to-beat arterial compliance and investigate its role in this entanglement of dynamical interactions. However, some nodes in the network are still missing which may play an important role as confounders. For example, it is relevant to mention the roles of stroke volume and cardiac output, which were cited in this work as a mean to either calculate beat-to-beat arterial compliance from the IMP waveform or discuss the results. In this regard, exploring extended networks involving more signals would be of interest for further studies, keeping in mind the already discussed limits that arise when working with high-dimensional systems, especially for the model-free approach. Nevertheless, it is worth mentioning the remarkable role of vasomotion, i.e., vasoconstriction and vasodilation phenomena due to sympathetic activity directed to the vessels, which may have an influence on arterial compliance [78,86]. Sympathetic activity thus is a common source of oscillations in arterial compliance, heart rate, PVR and BP - although the magnitudes of its influence on given measure can be relatively independent. This represents a valuable limitation in the study of physiological dynamics, as the sympathetic firing cannot be measured nor disregarded but undoubtedly has significant effects on the interplay among variables in the considered network. Among the approaches that could be employed to mitigate or quantify the role of possible confounders, it is possible to confine the analysis to specific spectral bands of interest, e.g., removing the high-frequency oscillatory content of the signals to eliminate the effect of respiration.

From a methodological perspective, it is important to highlight how, in contrast to other approaches such as those based on causal discovery or structure learning methods [87,88], the measures employed in this study are based on the observed behaviour and functionality of the investigated physiological signals, rather than on the presence or absence of structural mechanisms, a factor that could be of interest in order to achieve a deeper understanding of both physiology and anatomy underlying physiological networks. Moreover, it is important to highlight how the aforementioned approaches are mostly employed to investigate network involving random variables, while we focus on random processes presenting temporal correlations. Unlike approaches used to infer directionality and causality interactions within physiological networks, such as those based on phase-based bidirectional coupling measures [89,90], causality measures employed here are less computationally efficient in the case of nonlinear dynamics and require data stationarity. Notwithstanding, it is important to emphasize that the majority of available causality measures are bivariate, thus incapable of being extended to a conditional form necessary for the assessment of direct causal links, as is possible with the measures employed in this work. Nevertheless, although being multivariate, conditional causality measures have the main drawback of not taking into account the high-order effects of complex control mechanisms. It has been demonstrated that conditional transfer entropy may fail to reveal redundant effects among the observed processes [91]. Further analysis, based either on decomposing the information shared into unique, synergistic, and redundant contributions [92] or exploring the redundant/synergistic role of network links in information processing [22], should be carried on to achieve a more detailed description of the node-, link- and network-wise behaviors of the considered physiological network, as well as to better account for the aforementioned role of not easily measurable signals seen as confounders.

## Conclusion

This work shed light on the complex dynamics involved in the short-term cardiovascular regulation during the resting state and in response to postural stress in young healthy subjects employing solely noninvasive techniques. The rationale of our approach is that analyzing physiological networks with many nodes via the use of measures capable to capture directed interactions and account for possible confounders can better elicit the several mechanisms involving cardiovascular and cardiorespiratory regulation. The integration of arterial compliance variability within a four-node network including heart period, arterial pressure and respiration allowed a more complete description of cardiovascular and cardiorespiratory mechanisms. This approach favored the inference of the physiological network behind the homeostatic control of cardiac, vascular and respiratory activities, showing the ability of healthy subjects to respond adequately to postural stress and the associated modifications. Results confirmed already known physiological control mechanisms involving cardiovascular dynamics, e.g., the baroreflex loading, or cardiac and respiratory activities, e.g., the RSA mechanism, but also led to interpret less studied mechanisms involving, e.g., the influence of cardiac and blood pressure dynamics on arterial compliance, as well as the effect of compliance on breathing patterns.

Furthermore, the comparison between parametric and nonlinear model-free approaches for the inference of direct causality allows for a description of the linear vs. nonlinear physiological mechanisms involved in the resting state and during the postural stress. Among the several physiological interactions identified, our analyses show that cardiorespiratory interactions represent the strongest mechanism within the investigated network, and how this interplay involves nonlinear dynamics with relevant fast, within-beats effects. Furthermore, besides the well-known cardiac-respiratory influence on tilt-induced changes of blood pressure, our results evidenced the emergence of a likely nonlinear causal influence of heart period on arterial compliance and of linear bidirectional interactions involving compliance and mean arterial pressure dynamics. For the first time, we assessed the important role of arterial compliance within a complex network of cardiovascular variables, evidencing that short-term mechanisms affecting the beat-to-beat variability of this parameter are essentially within-beat and nonlinear. The latter is a novel observation which poses the basis for further and deeper investigation.
